# From Agent-Based Markov Dynamics to Hierarchical Closures on Networks: Emergent Complexity and Epidemic Applications

**DOI:** 10.3390/e28010063

**Published:** 2026-01-05

**Authors:** A. Y. Klimenko, A. Rozycki, Y. Lu

**Affiliations:** Centre for Multiscale Energy Systems, School of Mechanical and Mining Engineering, The University of Queensland, St. Lucia, Brisbane 4072, Australia; a.rozycki@uq.edu.au (A.R.); y.lu7@uq.edu.au (Y.L.)

**Keywords:** SIR epidemic, network clustering, BBGKY hierarchy, conditional moments

## Abstract

We explore a rigorous formulation of agent-based SIR epidemic dynamics as a discrete-state Markov process, capturing the stochastic propagation of infection or an invading agent on networks. Using indicator functions and corresponding marginal probabilities, we derive a hierarchy of evolution equations that resembles the classical BBGKY hierarchy in statistical mechanics. The structure of these equations clarifies the challenges of closure and highlights the principal problem of systemic complexity arising from stochastic but generally not fully chaotic interactions. Monte Carlo simulations are used to validate simplified closures and approximations, offering a unified perspective on the interplay between network topology, stochasticity, and infection dynamics. We also explore the impact of lockdown measures within a networked agent framework, illustrating how SIR dynamics and structural complexity of the network shape epidemic with propagation of the COVID-19 pandemic in Northern Italy taken as an example.

## 1. Introduction

Understanding the stochastic dynamics of epidemics, particularly those involving competitive propagation, remains a central challenge not only in epidemiology but also in related fields such as the spread of dominant species or technological innovations. The topology of the invaded space can often be effectively represented as a network, introducing additional complexity into the dynamics of the epidemic. The classical SIR (Susceptible/Infected/Recovered) model forms the basis for many such studies and has been extended to incorporate realistic transmission patterns using network-based formulations [1]. Clear specification and relative simplicity is a significant advantage of SIR as a standard model capturing principal physical processes. Agent-based models (ABMs) offer a powerful but computationally expensive framework for evaluating the overall dynamics based on individual-level interactions [2,3]—these models conceptually replicate particle approaches in the modelling of reacting flows [4].

In this work, we adopt a probabilistic and systemic perspective by modelling agent-based SIR dynamics as a continuous-time Markov process. Each individual is treated as a node in a graph, whose state evolves due to infection and recovery events governed by stochastic rules and controlled by the corresponding master (Kolmogorov) equations. This framework allows for a rigorous derivation of time-dependent joint and marginal probability distributions that describe the transmission.

Our formulation follows the physics-based approach of Omata [5], but departs from traditional moment-based closures by deriving the governing equations directly from the indicator functions associated with individual node states. Taking ensemble averages of products of these indicators yields exact evolution equations for marginal probabilities. The resulting structure forms an explicit and interpretable hierarchy: equations for low-order marginals depend on higher-order marginals because infection events couple the stochastic states of neighbouring nodes.

This hierarchy is closely related in spirit to the Bogoliubov–Born–Green–Kirkwood–Yvon (BBGKY) hierarchy in statistical mechanics [6,7,8,9]. In kinetic theory, the BBGKY hierarchy arises when the high-dimensional Liouville equation is reduced to one- or few-particle marginal distributions: interactions ensure that each reduced equation involves higher-order distribution functions and is therefore unclosed at any fixed level. Boltzmann’s closure becomes possible only under additional assumptions, most notably the molecular chaos (Stosszahlansatz) hypothesis [10,11]. A closely related issue arises in particle-based or agent-based models: low-order descriptions close easily only under “chaotic” assumptions, whereas departures from chaos correspond to the emergence of non-trivial correlations and, more broadly, to the emergence of complexity [12,13,14].

In the present work, rather than introducing heuristic closures at the outset, we retain the hierarchy in a symbolic and formally exact form, making the dependence on higher-order stochastic structure explicit. In contrast to many network–epidemic models based on expected values and deterministic ODEs, this formulation preserves the probabilistic content of the underlying continuous-time Markov jump process. Generalised derivatives provide a convenient calculus for jump processes (infection and recovery events) and connect naturally to ensemble-based Monte Carlo realisations [15], thereby unifying analytical derivations and numerical simulations within a single framework.

Finally, real epidemics and realistic contact structures involve interventions (e.g., lockdown-type reductions in transmission), behavioural adaptation, and pronounced network heterogeneity. To probe these effects within the same modelling framework, we examine how intervention timing and intensity interact with a clustered network structure to shape epidemic propagation. This complements recent work on nonlinear outcomes in temporal and adaptive networks [16,17] and highlights how structural constraints and stochastic transmission jointly govern multi-wave and long-tailed dynamics. Our main aim, however, is methodological: to model and analyse complex effects, rather than to deliver a comprehensive representation of a realistic epidemic.

Section 2 introduces the agent-based epidemic model as a continuous-time Markov process and defines its probabilistic structure using indicator functions and marginal distributions. Section 3 is dedicated to deriving the governing equations for fine-grained and marginal probabilities, revealing a BBGKY-like hierarchy. In Section 4, several closure strategies are proposed to make this hierarchy tractable. Section 5 benchmarks these closures against exact solutions and Monte Carlo simulations on simple graphs. Section 6 extends the analysis to randomly constructed networks, while Section 7 summarises the key findings. Appendix A provides an example application of the model, together with a nomenclature list.

## 2. Agent-Based Epidemic Modelling as a Markov Process

### 2.1. System States and Their Full Joint Probability Distribution

In general, agent-based models involve two principal categories of agents: nodes, which remain stationary, and particles, which can move from one node to another. Both categories of agents can possess properties that may evolve in time and/or change due to interactions with other agents. In this work, we focus on the interpretation of an epidemic model that represents individuals as nodes numbered i=1,2, …, N. Each node *i* has a property $Y_{i}$ that can take several values. According to the traditional SIR (susceptible, infected, recovered) model, $Y_{i}$ can take one of the values S, I, or R. Therefore the state of the system of nodes is given by the following vector:

(1) $$Y^{\left(N\right)}=\left[Y_{1},Y_{2},\ldots ,Y_{N}\right]\;.$$

For example, S_1_R_2_R_3_I_4_,…,$I_{N-1}$,$S_{N}$ is a possible state of the system, where nodes 1 and *N* are susceptible, nodes 4 and N−1 are infected, and nodes 2 and 3 have recovered. There are $3^{N}$ possible states for this system. While more sophisticated models, which, for example, may involve several infected states $I^{\left(1\right)},$$I^{\left(2\right)},\ldots$ can be formulated for specific diseases and our analysis can be easily extended to such models; we prefer to keep our consideration general and focus on complexity emerging at systemic levels. We take a systemic perspective and are interested in general conceptual properties rather than a detailed description of a specific infection.

The propagation of an epidemic is, evidently, a random process which can be characterised by the corresponding *joint probabilities* $P_{Y}^{\left(N\right)}=P\left(Y_{1}^{\circ },Y_{2}^{\circ },\ldots ,Y_{N}^{\circ }\right)$ that can be expressed as the following ensemble average:

(2) $$P_{Y}=P^{\left(N\right)}=P\left(Y_{1}^{\circ },Y_{2}^{\circ },\ldots ,Y_{N}^{\circ }\right)=\langle \theta_{1}\left(Y_{1}^{\circ }\right)\theta_{2}\left(Y_{2}^{\circ }\right)\ldots \theta_{N}\left(Y_{N}^{\circ }\right)\rangle$$

of the indicator functions

(3) $$\theta_{i}\left(Y^{\circ }\right)=\delta_{Y_{i}Y^{\circ }}=\left\{\begin{array}{cc} 1, & Y_{i}=Y^{\circ }\\ 0, & Y_{i}\neq Y^{\circ } \end{array}\right.\;.$$

Here, δ denotes the Kronecker delta, while $\theta_{i}\left(Y^{\circ }\right)$ is a stochastic function that depends on location *i* and the sample-space parameter $Y^{\circ },$ which can take one of the three values {S,I,R}. We use the superscript “∘” to distinguish a random value $Y_{i}\left(t\right),$ which is the actual state of the node *i* at a given time moment t, from the corresponding sample-space parameter $Y_{i}^{\circ },$ which does not depend on time and is an argument of the function $\theta_{i}\left(\ldots \right)$. For example, if $Y_{i}=$ I, then $\theta_{i}(I)=1$ and $\theta_{i}\left(S\right)$$=\theta_{i}(R)=0$. Note that the indicator functions depend on time $\theta_{i}\left(Y_{i}^{\circ }\right)=\theta_{i}\left(Y_{i}^{\circ },t\right)$ since $Y_{i}=Y_{i}\left(t\right)$ in definition (3). The complete probability function $P_{Y}$ depends on *N* sample-space parameters $Y_{1}^{\circ },Y_{2}^{\circ },\ldots ,Y_{N}^{\circ }$, and each of these parameters can independently take the three values: S, I, or R. Note that the order of the nodes 1,…,i,…,N is deemed to be fixed to avoid confusion.

### 2.2. Agent-Based Models as Networks

The SIR model involves interactions between individuals that propagate infection from one individual to another. Similar mechanisms are engaged in the transmission of ideas, news, or other types of information between individuals. These individuals are represented by nodes, which in addition to properties $Y_{i},$ are characterised by connections to other nodes, indicating possible routes for transmission of the infection (or information). Note that infection can propagate in both directions (i.e., from *i* to *j* and from *j* to *i* but only if nodes *i* and *j* are connected). Hence, from the mathematical perspective, the system of nodes is an undirected graph or network. The adjacency matrix associated with this graph is denoted by $A_{ij}$—this matrix has positive values if and only if i↔j (i.e., if nodes *i* and *j* are connected). Note that the adjacency matrix is symmetric $A_{ij}=A_{ji}$ for undirected graphs and, conventionally, $A_{ii}=0.$ Representing interactions between individuals by graphs is effective since each individual usually has relatively few direct contacts, while contacts with the rest of the population are absent or negligible. When using graphs, we avoid considering interactions between nodes that do not interact. Graphs and networks are characterised by the overall number of nodes *N* and the overall number of edges E. In real-world networks, the number of nodes *N* can reach millions, while the number of edges is much smaller than its maximal value $N\ll N_{\max}=n\left(n-1\right)/2$. Two classes of graphs can be considered: weighted and unweighted. For unweighted graphs, the nodes *i* and *j* are either connected $A_{ij}=1$ or not $A_{ij}=0$. In weighted graphs, each positive value $A_{ij}$ reflects the intensity of connections between nodes *i* and j:

(4) $$A_{ij}=A_{ji}=\left\{\begin{array}{cc} >0 & i↔j\;\\ =0 & i↮j \end{array}\right.,\;\;\;\;i,j=1,2,\ldots ,N\;.$$

While it is natural to use networks to represent contacts and communications between individuals, the properties of these networks evolved in modern society. While networks of the past were subject to localisation determined by physical distances, modern technology largely removed these constraints, allowing for effective communication and fast transportation. These modern networks have so-called small-world properties: the number of nodes $N_{r}$ located within distance *r* (measured in the minimal number of edges required to pass while moving from one node to another) increases exponentially with *r*

(5) $$N_{r}∼\exp\left(r\right),$$

which is much faster than, say, the estimate $N_{r}∼r^{2}$ that is valid for a network localised on a two-dimensional surface. The modern world is highly interconnected, creating favourable conditions not only for the exchange of knowledge and information but also for the spread of infections. Such spread remains, to a large extent, diffusive in character, being driven by a multitude of local contacts [18]. However, occasional long-distance “jumps” can substantially accelerate transmission, as illustrated by the small-world network phenomenon.

### 2.3. The Forward Kolmogorov Equation

From the perspective of the probability theory, the evolution of the system of nodes is a Markov chain. In simple terms, the Markov property implies that given the complete present state, we do not need to know the past to predict the future—this is a natural assumption used in this and many other applications. The system evolves by random transitions between states so that the evolution of the probabilities is described by the so-called direct Kolmogorov equation.

(6) $$\frac{dP_{Y^{\prime }}}{dt}=\sum_{Y^{\prime \prime }}{\bar{T}}_{Y^{\prime }\leftarrow Y^{\prime \prime }}P_{Y^{\prime \prime }}-\sum_{Y^{\prime \prime }}{\bar{T}}_{Y^{\prime \prime }\leftarrow Y^{\prime }}P_{Y^{\prime }},$$

where ${\bar{T}}_{Y^{\prime }\leftarrow Y^{\prime \prime }}$ denotes the average transition rates from state $Y^{\prime \prime }$ to state $Y^{\prime }$ and specify the transition coefficients of the equation. The first term in (6) evaluates all transitions into state $Y^{\prime }$ while the second term in (6) sums up all transitions from state $Y^{\prime }$. These two terms can be assembled into a single matrix ${\bar{\bar{T}}}_{Y^{\prime }Y^{\prime \prime }}$ so that

(7) $$\begin{array}{cc} \frac{dP_{Y}}{dt} & =\bar{\bar{T}}\cdot P_{Y}=\sum_{Y^{\prime \prime }}{\bar{\bar{T}}}_{Y^{\prime }Y^{\prime \prime }}P_{Y^{\prime \prime }},\;\; \end{array}$$

(8) $$\begin{array}{cc} \;\;{\bar{\bar{T}}}_{Y^{\prime }Y^{\prime \prime }} & ={\bar{T}}_{Y^{\prime }\leftarrow Y^{\prime \prime }}-\delta_{Y^{\prime }Y^{\prime \prime }}\sum_{Y^{\prime \prime \prime }}{\bar{T}}_{Y^{\prime \prime \prime }\leftarrow Y^{\prime }}\;. \end{array}$$

The matrix ${\bar{\bar{T}}}_{Y^{\prime }Y^{\prime \prime }}$ is conventionally called the transition rate matrix—operator ${\bar{\bar{T}}}_{Y^{\prime }Y^{\prime \prime }}$ is specified in the following sections. The dimension of this matrix, $3^{N}\times 3^{N}$, is determined by the overall number of states and only a small fraction of these values is non-zero. For the examples presented in this work, *N* is at least 500 and the full joint probability distribution $P_{Y}$ is represented by $3^{500}$ real numbers. Note that the value $3^{500}$ exceeds by far the number of elementary particles in the known universe (which is merely $10^{80}$). It is needless to say that solving such a large number of equations is completely impossible, even if we can scrupulously specify all transition coefficients. Therefore, one needs to consider possible simplifications.

### 2.4. Marginal Probabilities

The problem becomes more traceable if expressed in terms of the marginal probabilities

(9) $$P^{\left(n\right)}=P\left(Y_{i_{1}}^{\circ },Y_{i_{2}}^{\circ },\ldots ,Y_{i_{n}}^{\circ }\right)=\langle f^{\left(n\right)}\rangle =\langle \theta_{i_{1}}\left(Y_{i_{1}}^{\circ }\right)\theta_{i_{2}}\left(Y_{i_{2}}^{\circ }\right)\ldots \theta_{i_{n}}\left(Y_{i_{n}}^{\circ }\right)\rangle ,$$

where n≤N and the set $i_{1},i_{2},\ldots ,i_{n}$ is a subset of length *n* of the overall set of nodes 1,2,…,N. Note that $i_{1},i_{2},\ldots ,i_{n}$ is not a fixed particular set (say, the set of 1,2,…,n) but reflects all possible choices of *n* elements from the full set 1,2,…,N of *N* elements. Using ensemble averages in (9) immediately tells us that the value of $P^{\left(n\right)}$ does not depend on the order of the arguments, that is $P\left(Y_{i_{1}}^{\circ },Y_{i_{2}}^{\circ },\ldots ,Y_{i_{n}}^{\circ }\right)$ is the same for any permutation of $Y_{i_{1}}^{\circ },Y_{i_{2}}^{\circ },\ldots ,Y_{i_{n}}^{\circ }$. For example, $P\left(Y_{1}^{\circ },Y_{2}^{\circ }\right)=P\left(Y_{2}^{\circ },Y_{1}^{\circ }\right).$ The product $f^{\left(n\right)}=\theta_{i_{1}}\left(Y_{i_{1}}^{\circ }\right)\ldots \theta_{i_{n}}\left(Y_{i_{n}}^{\circ }\right)$ is often called the fine-grained distribution and its average is the corresponding probability distribution $P^{\left(n\right)}=\langle f^{\left(n\right)}\rangle$. If n=N, then $P^{\left(N\right)}=P_{Y}$ represents the full joint probability. Since, obviously

$$\theta_{i}\left(Y^{\prime }\right)\theta_{i}\left(Y^{\prime \prime }\right)=\left\{\begin{array}{cc} \theta_{i}\left(Y^{\prime }\right), & Y^{\prime }=Y^{\prime \prime }\\ 0, & Y^{\prime }\neq Y^{\prime \prime } \end{array}\right.$$

the repeated nodes can be eliminated

(10) $$P\left(Y_{i_{1}}^{\circ },\ldots ,Y_{j}^{\prime },\ldots ,Y_{j}^{\prime \prime }\ldots ,Y_{i_{n}}^{\circ }\right)=P\left(Y_{i_{1}}^{\circ },\ldots ,Y_{j}^{\prime },\ldots ,Y_{i_{n}}^{\circ }\right)\delta_{Y_{j}^{\prime }Y_{j}^{\prime \prime }}\;.$$

We do not need to consider any distributions with n>N since the repeated nodes can always be eliminated according to Equation (10).

The one-node (or first-order) probability distributions $P^{\left(1\right)}=P_{Y_{i}}=P\left(Y_{i}^{\circ }\right)=\langle \theta_{i}\left(Y_{i}^{\circ }\right)\rangle$ specify the probability of node *i* being in a particular state $Y_{i}^{\circ }$ (denoting one of S, I, or R). These values, $P\left(Y_{i}^{\circ }\right),$ characterise only 3N states (i.e., three states for every node i=1,…,N). In the same way, the two-node probabilities $P^{\left(2\right)}=P_{Y_{i}Y_{j}}=P\left(Y_{i}^{\circ },Y_{j}^{\circ }\right)=\langle \theta_{i}\left(Y_{i}^{\circ }\right)\theta_{j}\left(Y_{j}^{\circ }\right)\rangle$ are characterised by 3N×3N real values specifying probabilities for all possible choices of nodes *i* and *j*. When this cannot cause ambiguity, the subscript is used to indicate the random variables that are characterised by *P*. For example, $P_{Y_{i}Y_{j}}$ is the marginal joint probability distribution of $Y_{i}$ and $Y_{j},$ and this distribution is a function of two sample-space arguments $P\left(Y_{i}^{\circ },Y_{j}^{\circ }\right)$. Note that any marginal probability $P^{\left(n\right)}$ can be conventionally expressed in terms of the full joint probability $P_{Y}$:

(11) $$P\left(Y_{1}^{\circ },Y_{2}^{\circ },\ldots ,Y_{n}^{\circ }\right)=\int P\left(Y_{1}^{\circ },Y_{2}^{\circ },\ldots ,Y_{n}^{\circ },Y_{n+1}^{\circ },\ldots ,Y_{N}^{\circ }\right)dY_{n+1}^{\circ }\ldots dY_{N}^{\circ }$$

or, similarly, in terms of marginal probabilities of $P^{\left(n^{\prime }\right)}$ of a higher order $n^{\prime }>n$.

## 3. The Governing Equations

### 3.1. Equations for the Fine-Grained Distributions

Deriving equations for the full and marginal probabilities needs clear notations and some care due to the large dimensions of the system under consideration. It seems that following effective techniques introduced in conditional methods [19] and using fine-grained distributions is one of the best possible choices. This approach is based on the following identity:

(12) $$\frac{df^{\left(n\right)}}{dt}=\frac{d\left(\theta_{i_{1}}\left(Y_{i_{1}}^{\circ }\right)\ldots \theta_{i_{n}}\left(Y_{i_{n}}^{\circ }\right)\right)}{dt}=\sum_{j=1}^{n}\left(\theta_{i_{1}}\left(Y_{i_{1}}^{\circ }\right)\ldots \left[\frac{d\theta_{i_{j}}\left(Y_{i_{j}}^{\circ }\right)}{dt}\right]\ldots \theta_{i_{n}}\left(Y_{i_{n}}^{\circ }\right)\right),$$

which, after averaging, results in

(13) $$\frac{dP^{\left(n\right)}}{dt}=\frac{d\langle f^{\left(n\right)}\rangle }{dt}=\langle \frac{df^{\left(n\right)}}{dt}\rangle =\sum_{j=1}^{n}\langle \theta_{i_{1}}\left(Y_{i_{1}}^{\circ }\right)\ldots \left[\frac{d\theta_{i_{j}}\left(Y_{i_{j}}^{\circ }\right)}{dt}\right]\ldots \theta_{i_{n}}\left(Y_{i_{n}}^{\circ }\right)\rangle \;.$$

Note that, since function $\theta_{i}\left(\ldots \right)$ takes discrete values 0 and 1, its conventional derivative does not exist and we customarily imply generalised derivatives. The full treatment of this problem is given in relevant textbooks and Ref. [19], but we can simply use formal differentiation rules since all these singularities disappear after averaging. We just need to evaluate $d\theta_{i}/dt$ for the SIR model. This model is characterised by two possible types of transitions—infection Φ and recovery Ψ—so that

(14) $$S\overset{\Phi }{⟶}I\overset{\Psi }{⟶}R\;.$$

If $\Phi_{i}$ is an instance of infection of node *i* from, say, node j, and $\Psi_{i}$ denotes an instance of recovery of node i, then $\Phi_{i}$ and $\Psi_{i}$ correspond to the following instantaneous transitions:

(15) $$\Phi_{i}=\sum_{j}T_{I_{i}I_{j}\leftarrow S_{i}I_{j}}=\sum_{j}p_{i}A_{ij}\theta_{i}\left(S\right)\theta_{j}\left(I\right),\;\;\;\Psi_{i}=T_{R_{i}\leftarrow I_{i}}=q_{i}\theta_{i}\left(I\right),$$

where $A_{ij}$ is the adjacency matrix determining connectivity between the nodes, $p_{i}$ specifies the probability of infection at node i, and $q_{i}$ specifies the probability of recovery of this node. As indicated in Equation (15), infection $T_{I_{i}I_{j}\leftarrow S_{i}I_{j}}$ is possible only when $Y_{i}=$ S and $Y_{j}=$ I, while recovery $T_{R_{i}\leftarrow I_{i}}$ requires that $Y_{i}=$ I. Note that transitions at nodes *i* and *j* depend on $Y_{i}$ and $Y_{j},$ and do not directly depend on the states of the other nodes.

Equations (14) and (15) determine that

(16) $$\frac{d\theta_{i}\left(S\right)}{dt}=-\delta_{\phi }\Phi_{i},\;\;\;\frac{d\theta_{i}\left(I\right)}{dt}=\delta_{\phi }\Phi_{i}-\delta_{\psi }\Psi_{i},\;\;\;\;\frac{d\theta_{i}\left(R\right)}{dt}=\delta_{\psi }\Psi_{i}\;.$$

Here, the Delta-functions $\delta_{\phi }=\delta \left(t-t_{\phi }\right)$ and $\delta_{\psi }=\delta \left(t-t_{\psi }\right)$ are used to indicate the presence of singularities in the derivatives of the indicator functions θ(…), pointing to jumps at random time moments: the instant of infection $t_{\phi }$ or the instant of recovery $t_{\psi }$. Equation (16) involves unit jumps indicated by the Delta-functions and the rates of these jumps determined by $\Phi_{i}$ and $\Psi_{i}$. For our purposes, the Delta-functions can simply be omitted in all equations, since $\delta_{\phi }$ and $\delta_{\psi }$ disappear after averaging and do not affect the final equations—we retain these terms only for the sake of rigour. With the use of the following indicator functions

(17) $$\phi \left(Y^{\circ }\right)=\left\{\begin{array}{cc} -1, & Y^{\circ }=S\\ +1, & Y^{\circ }=I\\ 0, & Y^{\circ }=R \end{array}\right.,\;\;\;\psi \left(Y^{\circ }\right)=\left\{\begin{array}{cc} 0, & Y^{\circ }=S\\ -1, & Y^{\circ }=I\\ +1, & Y^{\circ }=R \end{array}\right.,$$

Equations (14)–(16) can be written as

(18) $$\frac{d\theta_{i}\left(Y^{\circ }\right)}{dt}=\delta_{\psi }q_{i}\psi \left(Y^{\circ }\right)\theta_{i}\left(I\right)+\delta_{\phi }p_{i}\phi \left(Y^{\circ }\right)\sum_{j}A_{ij}\theta_{i}\left(S\right)\theta_{j}\left(I\right)$$

where $Y^{\circ }$ can take any of S, I, or R. The substitution of (18) into (12) yields the evolution equation for the fine-grained distribution:

(19) $$\begin{array}{cc} \frac{df^{\left(n\right)}}{dt} & =\frac{d\theta_{i_{1}}\left(Y_{i_{1}}^{\circ }\right)\ldots \theta_{i_{n}}\left(Y_{i_{n}}^{\circ }\right)}{dt}\\ \; & =\sum_{j=1}^{n}\left(\theta_{i_{1}}\left(Y_{i_{1}}^{\circ }\right)\ldots \left[\delta_{\psi }q_{i_{j}}\psi \left(Y_{i_{j}}^{\circ }\right)\theta_{i_{j}}\left(I\right)+\delta_{\phi }p_{i_{j}}\phi \left(Y_{i_{j}}^{\circ }\right)\sum_{k}A_{i_{j}k}\theta_{i_{j}}\left(S\right)\theta_{k}\left(I\right)\right]\ldots \theta_{i_{n}}\left(Y_{i_{n}}^{\circ }\right)\right) \end{array}$$

### 3.2. Equations for Marginal Probabilities

The governing equation for the marginal probabilities is the ensemble average of Equation (19).

(20) $$\begin{array}{cc} \; & \frac{dP\left(Y_{i_{1}}^{\circ },Y_{i_{2}}^{\circ },\ldots ,Y_{i_{n}}^{\circ }\right)}{dt}\\ \; & =\sum_{j=1}^{n}\left[q_{i_{j}}\psi \left(Y_{i_{j}}^{\circ }\right)P\left(Y_{i_{1}}^{\circ },\ldots ,I_{i_{j}},\ldots ,Y_{i_{n}}^{\circ }\right)+p_{i_{j}}\phi \left(Y_{i_{j}}^{\circ }\right)\sum_{i_{n+1}}A_{i_{j}i_{n+1}}P\left(Y_{i_{1}}^{\circ },\ldots ,S_{i_{j}},\ldots ,Y_{i_{n}}^{\circ },I_{i_{n+1}}\right)\right]\;. \end{array}$$

Since this equation is quite general but cumbersome, we also give the first- and second-order equations—specific forms of Equation (20) for one-node $P^{\left(1\right)}=P_{Y_{i}}=P\left(Y_{i}^{\circ }\right)$ and two-node $P^{\left(2\right)}=P_{Y_{i}Y_{j}}=P\left(Y_{i}^{\circ },Y_{j}^{\circ }\right)$ probability distributions.

#### 3.2.1. The First-Order Equations

At the *first order*, we obtain

(21) $$\frac{dP_{S_{i}}}{dt}=-{\bar{\Phi }}_{i},\;\;\;\;\frac{dP_{I_{i}}}{dt}={\bar{\Phi }}_{i}-{\bar{\Psi }}_{i},\;\;\;\;\frac{dP_{R_{i}}}{dt}={\bar{\Psi }}_{i},$$

where

(22) $${\bar{\Phi }}_{i}=\langle \Phi_{i}\rangle =p_{i}\sum_{j}A_{ji}P_{I_{j}S_{i}},\;\;{\bar{\Psi }}_{i}=\langle \Psi_{i}\rangle =q_{i}P_{I_{i}}$$

denote the average rates of infection and recovery. These equations for one-node probability distributions $P_{S_{i}},$ $P_{I_{i}}$ and $P_{R_{i}}$ also involve two-node probability $P_{I_{j}S_{i}}=P_{S_{i}I_{j}}$.

#### 3.2.2. The Second-Order Equations

At the *second order*, the equations for two-node probabilities are obtained by substituting n=2 into (20) or, equivalently, by averaging (12) and (16) for n=2 and producing the following set of equations:

(23) $$\begin{array}{ccc} \frac{dP_{S_{i}S_{j}}}{dt}=-{\bar{\Phi }}_{iS_{j}}-{\bar{\Phi }}_{jS_{i}}, & \frac{dP_{I_{i}S_{j}}}{dt}={\bar{\Phi }}_{iS_{j}}-{\bar{\Phi }}_{jI_{i}}-{\bar{\Psi }}_{iS_{j}}, & \frac{dP_{R_{i}S_{j}}}{dt}={\bar{\Psi }}_{iS_{j}}-{\bar{\Phi }}_{jR_{i}},\\ \frac{dP_{S_{i}I_{j}}}{dt}={\bar{\Phi }}_{jS_{i}}-{\bar{\Phi }}_{iI_{j}}-{\bar{\Psi }}_{jS_{i}}, & \frac{dP_{I_{i}I_{j}}}{dt}={\bar{\Phi }}_{iI_{j}}-{\bar{\Psi }}_{iI_{j}}+{\bar{\Phi }}_{jI_{i}}-{\bar{\Psi }}_{jI_{i}}, & \frac{dP_{R_{i}I_{j}}}{dt}={\bar{\Psi }}_{iI_{j}}+{\bar{\Phi }}_{jR_{i}}-{\bar{\Psi }}_{jR_{i}},\\ \frac{dP_{S_{i}R_{j}}}{dt}={\bar{\Psi }}_{jS_{i}}-{\bar{\Phi }}_{iR_{j}}, & \frac{dP_{I_{i}R_{j}}}{dt}={\bar{\Psi }}_{jI_{i}}+{\bar{\Phi }}_{iR_{j}}-{\bar{\Psi }}_{iR_{j}}, & \frac{dP_{R_{i}R_{j}}}{dt}={\bar{\Psi }}_{iR_{j}}+{\bar{\Psi }}_{jR_{i}}, \end{array}$$

where we denote ${\bar{\Phi }}_{iY_{j}}=\langle \Phi_{i}\theta \left(Y_{j}\right)\rangle$ and ${\bar{\Psi }}_{iY_{j}}=\langle \Psi_{i}\theta \left(Y_{j}\right)\rangle$ so that

(24) $$\begin{array}{ccc} {\bar{\Phi }}_{iS_{j}}=p_{i}\sum_{k\neq j}A_{ki}P_{I_{k}S_{i}S_{j}} & {\bar{\Phi }}_{iI_{j}}=p_{i}\sum_{k}A_{ki}P_{I_{k}S_{i}I_{j}} & {\bar{\Phi }}_{iR_{j}}=p_{i}\sum_{k\neq j}A_{ki}P_{I_{k}S_{i}R_{j}}\\ {\bar{\Psi }}_{iS_{j}}=q_{i}P_{I_{i}S_{j}} & {\bar{\Psi }}_{iI_{j}}=q_{i}P_{I_{i}I_{j}} & {\bar{\Psi }}_{iR_{j}}=q_{i}P_{I_{i}R_{j}} \end{array}\;.$$

The matrix in (23) is symmetric (that is $P\left(Y_{i}^{\prime },Y_{j}^{\prime \prime }\right)=P\left(Y_{j}^{\prime \prime },Y_{i}^{\prime }\right)$ but, generally, $P\left(Y_{i}^{\prime },Y_{j}^{\prime \prime }\right)\neq P\left(Y_{i}^{\prime \prime },Y_{j}^{\prime }\right)$), involving only six independent equations. Since the one-node probabilities can be obtained from two-node probabilities, Equations (21) and (22) do not generally need to be solved in conjunction with Equations (23) and (24). The second order system, however, is not closed since the equations for two-node probabilities involve the following three-node probabilities: $P_{S_{i}I_{k}S_{j}},$ $P_{S_{i}I_{k}I_{j}}$, and $P_{S_{i}I_{k}R_{j}}$. Some terms with k=j are excluded from the sums in (24) since $P_{S_{i}I_{j}S_{j}}=P_{S_{i}I_{j}R_{j}}=0$ according to (10). While Equations (23) and (24) are generally valid for any choice of nodes *i* and *j*, we need to consider only the connected nodes, i.e., nodes *i* and *j* ensure that $A_{ij}>0$. Hence, node *i* is connected with both node *k* and node *j* in the three-node probabilities $P_{I_{k}S_{i}Y_{j}}$ that are summated in (24). The overall number of equations is of the order $∼3^{2}E$, where *E* is the number of edges in the graph.

### 3.3. Conceptual Interpretation of the Governing Equations

One can note that the number of equations rapidly increases ∼3^*n*^ with the order of the system, but the system of equations remains unclosed. Indeed, Equation (20) has the functional form of

(25) $$\frac{dP^{\left(n\right)}}{dt}={\bar{\bar{T}}}_{\psi }^{\left(n\right)}\cdot P^{\left(n\right)}+{\bar{\bar{T}}}_{\phi }^{\left(n\right)}\cdot P^{\left(n+1\right)},$$

so that the governing equations for $P^{\left(n\right)}$ involve $P^{\left(n+1\right)}$, while the governing equations for $P^{\left(n+1\right)}$ involve $P^{\left(n+2\right)}$ and so on until n=N is reached. Here, ${\bar{\bar{T}}}_{\psi }^{\left(n\right)}$ and ${\bar{\bar{T}}}_{\phi }^{\left(n\right)}$ denote linear operators (transitional matrices) that reflect transitions correspondingly associated with recovery and infection; these operators are specified by the two terms on the right-hand side of Equation (20). Since any probability $P^{\left(N+1\right)}$ must have repeated nodes and, as noted in (10), can be expressed in terms of $P^{\left(N\right)},$ Equation (20) becomes

(26) $$\frac{dP^{\left(N\right)}}{dt}={\bar{\bar{T}}}_{\psi }^{\left(N\right)}\cdot P^{\left(N\right)}+{\bar{\bar{T}}}_{\phi }^{\left(N\right)}\cdot P^{\left(N\right)}$$

for n=N. Unlike (25), this equation is closed and, of course, coincides with the forward Kolmogorov Equation (6) that gives a complete description for the whole system of *N* nodes.

While Equation (25) can be solved for small values of *n*, these equations are not closed and force us to consider higher and higher orders *n*. Equation (26) is closed but is practically unsolvable due to its extremely large dimensionality. This is not accidental—similar problems are known to exist in large and complex systems including multi-particle quantum mechanics and statistical physics. Equation (26) is similar to the Liouville equation of statistical physics—both equations are exact and useless for simulations due to their extremely large dimensionality. Equation (25) resembles the BBGKY (Bogoliubov–Born–Green–Kirkwood–Yvon) hierarchy, which involves unclosed equations [9,12]. The practical way of solving such equations is by applying the hypothesis of molecular chaos and decoupling distributions—this procedure results in the Boltzmann equation leading to the famous H-theorem. Similar problems can be found in general particle modelling associated with reacting flows, producing a hierarchy of equations of increasing dimensionality. At the systemic level, there is a great deal of similarity between all these problems.

While we also use “chaotic decoupling” in this work, its application at the first order, as carried out in the conventional derivation of the Boltzmann equation, tends to produce inaccurate results. The systems we consider are not fully chaotic and, as known from publications [12,13,14], this is the first sign of emerging complexity. In complex systems, interactions between elements lead to substantial dependencies between them, violating “chaotic assumptions” and forcing us to consider multi-particle, multinode, and multivariable distributions.

### 3.4. Monte Carlo Simulations

If the evaluation of the probability distributions is difficult or impossible, one of the common solutions is resorting to Monte Carlo simulations, which direct emulations of the underlying stochastic processes. Typically Monte Carlo simulations are more computationally expensive than low-order distribution models but are much more affordable in comparison with solving equations for full joint distributions. As with any modelling method, Monte Carlo simulations have their pluses and minuses. In the context of the network SIR model, the Markov chain model is specified for a sufficiently small time step Δt and every node i=1,…,N by the following transitions:

(27) $$\begin{array}{cc} S_{i} & ⟶I_{i}\;\mathrm{with}\;\mathrm{the}\;\mathrm{probability}\;p_{\Delta t}=p_{i}\Delta t\sum_{j}A_{ij}\theta_{j}\left(I\right), \end{array}$$

(28) $$\begin{array}{cc} I_{i} & ⟶R_{i}\;\mathrm{with}\;\mathrm{the}\;\mathrm{probability}\;q_{\Delta t}=q_{i}\Delta t\;. \end{array}$$

The numerical issues are discussed further in the simulation section.

While using stochastic simulations, we still wish to obtain typical or average characteristics, which may be problematic. First, since epidemics are fundamentally unsteady processes, time averaging is not suitable for them. We, however, may try to average over nodes, assuming that the network does not have a strong localisation in the physical space associated with spatial inhomogeneity. This averaging may work as long as values at different nodes are not correlated, which, as noted above, is generally not correct. In the present work, we combine averaging over nodes with ensemble averaging; that is simulations are run independently many times and then average characteristics are evaluated. This increases expenses associated with Monte Carlo simulations.

There is another problem associated with stochastic simulations: real-world systems may involve ∼10^6^ elements (individuals) while we might use a graph of ∼10^3^ nodes to run simulations. The question of scaling up is not trivial. One issue is preserving the node degree distribution (which significantly affects simulations) when scaling networks—this issue is discussed further in the simulation section. The other issue is the possibility of global and local extinctions, which, as we know from the simulations of reacting flows, makes modelling complicated. Extinctions occur when nodes (individuals) recover before transferring the infection. The case of the reproduction number being close to unity is most complicated since the process may or may not become extinct depending on realisations. Since each node in simulations effectively represents a thousand individuals, it is clear that extinctions between a few elements are more probable than among thousands of individuals under the same conditions.

## 4. Closures for Marginal Distributions

### 4.1. The First-Order Closure

In this section, we conceptually follow Boltzmann’s hypothesis of molecular chaos that allows for the representation of two-particle distributions as the product of the corresponding one-particle distributions. In the context of the first-order system, which needs a closure for $P_{I_{j}S_{i}},$ this implies that

(29) $$P_{I_{j}S_{i}}=\left\{\begin{array}{cc} P_{I_{j}}P_{S_{i}}, & i\neq j\\ 0, & i=j \end{array}\right.$$

the two-node distribution $P_{I_{j}S_{i}}$ is assumed to be a product of the one-node distributions $P_{I_{j}}$ and $P_{S_{i}}$ implementing simple unconditional decoupling. Note that $P_{I_{i}S_{i}}$ does not enter Equation (22) since $A_{ii}=0$ and does not need to be specified; therefore, assuming $P_{I_{j}S_{i}}=P_{I_{j}}P_{S_{i}}$ for all *i* and *j* yields exactly the same model. For the sake of simplicity, the approximation details that do not affect the model are omitted from further consideration.

Substitution of the first-order decoupling closure (29) into Equations (21) and (22) results in the closed system for one-node probability distributions:

(30) $$\frac{dP_{S_{i}}}{dt}=-{\bar{\Phi }}_{i},\;\;\;\;\frac{dP_{I_{i}}}{dt}={\bar{\Phi }}_{i}-{\bar{\Psi }}_{i},\;\;\;\;\frac{dP_{R_{i}}}{dt}={\bar{\Psi }}_{i},$$

(31) $${\bar{\Phi }}_{i}=\sum_{j}p_{i}A_{ij}P_{I_{j}}P_{S_{i}},\;\;\;\;{\bar{\Psi }}_{i}=q_{i}P_{I_{i}},$$

where i=1,…,N. The first-order model involves only 3N ordinary differential equations but, as shown in the following sections, the first-order decoupling is not particularly accurate due to stochastic dependencies between neighbouring nodes.

### 4.2. The Ergodic Closure

This closure is suitable when the adjacency matrix is decomposed into two terms $A_{ij}=A_{ij}^{\circ }+A_{ij}^{\prime }$ so that the principal term has relatively few significant connections $A_{ij}^{\circ }∼1,$ while the second term reflects the possibility of numerous but weak (or occasional) connections $A_{ij}^{\prime }\ll 1$. The second term either can be negligible or may contribute to the overall evolution of the epidemic despite $A_{ij}^{\prime }\ll 1$ due to a large number of possible contacts. This contribution can be evaluated assuming that $A_{ij}^{\prime }=\varepsilon \ll 1$ is the same for all nodes (since its effect is averaged over a very large number of possible contacts), leading to Equation (22) taking the following form:

(32) $${\bar{\Phi }}_{i}=p_{i}\sum_{j}A_{ji}^{\circ }P_{I_{j}S_{i}}+p_{i}\varepsilon N{\bar{P}}_{{\mathrm{IS}}_{i}},\;\;\;\;{\bar{P}}_{{\mathrm{IS}}_{i}}=\frac{1}{N}\sum_{j}P_{I_{j}S_{i}}\;,$$

The first term is subject to the first- and second-order closures discussed in this section, while the node average probability is evaluated as

(33) $${\bar{P}}_{{\mathrm{IS}}_{i}}=\frac{1}{N}\sum_{j}\langle \theta_{j}\left(I\right)\theta_{i}\left(S\right)\rangle =\langle \theta_{i}\left(S\right)P_{I}\rangle =P_{S_{i}}P_{I}\;,$$

where the ergodic hypothesis

(34) $$\theta \left(I\right)\overset{\mathrm{def}}{=}\frac{1}{N}\sum_{j}\theta_{j}\left(I\right)\approx P_{I}\overset{\mathrm{def}}{=}\frac{1}{N}\sum_{j}P_{I_{j}}$$

is applied, implying that the average over all nodes coincides with the corresponding ensemble average. While decoupling (31) may or may not be accurate when applied to the principal part of the graph $A_{ij}^{\circ }$ requiring higher-order closures, decoupling (33) applied to secondary connections is much better since the states of weakly connected nodes are not likely to be strongly correlated. Yet the ergodic hypothesis is not exact, especially when extinctions are present. Indeed, by definition θ(I)=0 for extinct realisations, while $P_{I}>0$ when some of the realisations are not extinct. As in modelling of reacting flows, extinctions tend to increase systemic complexity.

In the simulations presented in this work, we do not consider secondary connections, assuming that $A_{ij}=1$ for connected nodes but, in the real world, occasional transmissions which have very low probability for given *i* and *j* may contribute significantly when the population is large N≫1.

### 4.3. Second-Order Direct Decoupling Closure

Second-order closure implies that $P_{S_{i}I_{j}}$ is not approximated by (29) but modelled using Equations (23) and (24). The second-order equation for $P_{I_{j}S_{i}}$

(35) $$\frac{dP_{I_{j}S_{i}}}{dt}=-p_{i}\sum_{k}A_{ki}P_{I_{k}S_{i}I_{j}}+p_{j}\sum_{k}A_{kj}P_{I_{k}S_{j}S_{i}}-q_{j}P_{I_{j}S_{i}}$$

remains unclosed due to the presence of the three-node probabilities $P_{I_{k}S_{i}I_{j}}$ and $P_{I_{k}S_{j}S_{i}}$, which need to be approximated. Note that although Equation (35) is valid for any i,j ∈1,…,N, we need to evaluate $P_{I_{j}S_{i}}$ only when $A_{ji}>0,$ i.e., for distinct connected nodes *i* and *j*. The following unconditional approximations

(36) $$P_{I_{k}S_{i}I_{j}}=\left\{\begin{array}{cc} P_{I_{k}S_{i}}P_{I_{j}}, & k\neq j\\ P_{I_{j}S_{i}}, & k=j \end{array}\right.,\;\;\;\;P_{I_{k}S_{j}S_{i}}=\left\{\begin{array}{cc} P_{I_{k}S_{j}}P_{S_{i}}, & k\neq i\\ 0, & k=i \end{array}\right.$$

lead to the system

(37) $$\frac{dP_{S_{i}}}{dt}=-{\bar{\Phi }}_{i},\;\;\;\;\frac{dP_{I_{i}}}{dt}={\bar{\Phi }}_{i}-{\bar{\Psi }}_{i},\;\;\;\;\frac{dP_{R_{i}}}{dt}={\bar{\Psi }}_{i}\;,$$

(38) $${\bar{\Phi }}_{i}=p_{i}\sum_{j}A_{ij}P_{I_{j}S_{i}},\;\;\;\;{\bar{\Psi }}_{i}=q_{i}P_{I_{i}}\;,$$

(39) $$\frac{dP_{I_{j}S_{i}}}{dt}=-p_{i}\sum_{k}A_{ki}P_{I_{k}S_{i}}P_{I_{j}}-\underset{\left(a\right)}{\underset{︸}{p_{i}A_{ji}P_{I_{j}S_{i}}\left(1-P_{I_{j}}\right)}}+p_{j}\sum_{k}A_{kj}P_{I_{k}S_{j}}P_{S_{i}}-\underset{\left(b\right)}{\underset{︸}{p_{j}A_{ij}P_{I_{i}S_{j}}P_{S_{i}}}}-q_{j}P_{I_{j}S_{i}}\;,$$

which is a closed system of 4N differential equations.

If more simple closures $P_{I_{k}S_{i}I_{j}}=P_{I_{k}S_{i}}P_{I_{j}}$ and $P_{I_{k}S_{j}S_{i}}=P_{I_{k}S_{j}}P_{S_{i}}$ for all i,j,k are used instead of (36), then terms (a) and (b) vanish from Equation (39). These simple closures are obviously incorrect since $P_{I_{j}S_{i}I_{j}}=P_{I_{j}S_{i}}\neq P_{I_{j}S_{i}}P_{I_{j}}$ and $P_{I_{i}S_{j}S_{i}}=0\neq P_{I_{i}S_{j}}P_{S_{i}}$ according to (10). Equation (39) can be compared with the equation

(40) $$\frac{dP_{I_{j}}P_{S_{i}}}{dt}=-p\sum_{k}A_{ki}P_{I_{k}S_{i}}P_{I_{j}}+p\sum_{k}A_{kj}P_{I_{k}S_{j}}P_{S_{i}}-qP_{I_{j}}P_{S_{i}}$$

for the product $P_{I_{j}}P_{S_{i}}$ obtained from (21) and (22). It is easy to see that Equation (40) coincides with Equation (39) whenever terms (a) and (b) are removed. This implies that, without the effects of terms (a) and (b), $P_{I_{j}S_{i}}=P_{I_{j}}P_{S_{i}}$ and the second-order model is functionally reduced to the first order.

### 4.4. Second-Order Conditional Closure

In addition to Equation (35), this closure uses another second-order equation

(41) $$\frac{dP_{S_{j}S_{i}}}{dt}=-p_{j}\sum_{k}A_{kj}P_{I_{k}S_{j}S_{i}}-p_{i}\sum_{k}A_{ki}P_{I_{k}S_{i}S_{j}}$$

for the two-node joint probability $P_{S_{j}S_{i}}$ obtained in (23) and (24). Both Equations (35) and (41) need closures for three-node probabilities $P_{I_{k}S_{j}S_{i}}$ and $P_{I_{k}S_{i}S_{j}}$, which is based on the following transformations, $P_{I_{k}S_{i}I_{j}}=P_{I_{k}I_{j}|S_{i}}P_{S_{i}}$ and $P_{I_{k}S_{i}S_{j}}=P_{I_{k}S_{j}|S_{i}}P_{S_{i}}$, where the vertical bar denotes conditional probabilities; for example, the probability $P_{I_{k}S_{j}|S_{i}}=P(I_{k}S_{j}|S_{i})$ is conditioned on $Y_{i}=S_{i}$. Note that it is the central node *i* which is connected by the graph to its neighbouring nodes *j* and *k* (so that $A_{ij}>0$ and $A_{ik}>0)$ that is selected for conditioning. The conditional closure is based on the following decoupling:

(42) $$P_{I_{k}S_{j}|S_{i}}=P_{I_{k}|S_{i}}P_{S_{j}|S_{i}}\;\;\;\mathrm{and}\;\;\;P_{I_{k}I_{j}|S_{i}}=P_{I_{k}|S_{i}}P_{I_{j}|S_{i}}\;.$$

While these relations are approximate, it is well-known that conditional decoupling implemented in conditional methods (e.g., Conditional Moment Closure and Multiple Mapping Conditioning—effective models used in simulations of reacting flows) is much better than any analogous unconditional decoupling. We also need to note the following identities:

(43) $$P_{I_{j}I_{j}|S_{i}}=P_{I_{j}|S_{i}}\;\;\;\mathrm{and}\;\;\;P_{I_{j}S_{j}|S_{i}}=0\;,$$

and obtain the relations

(44) $$P_{I_{k}S_{i}I_{j}}=\left\{\begin{array}{cc} P_{I_{k}|S_{i}}P_{S_{i}}P_{I_{j}|S_{i}}, & k\neq j\\ P_{I_{j}S_{i}}, & k=j \end{array}\right.,\;\;\;\;P_{I_{k}S_{i}S_{j}}=\left\{\begin{array}{cc} P_{I_{k}|S_{i}}P_{S_{i}}P_{S_{j}|S_{i}} & k\neq j\\ 0, & k=j \end{array}\right.\;,$$

which consistently implement conditional decoupling. The conditional closure results in the following system of equations:

(45) $$\frac{dP_{S_{i}}}{dt}=-{\bar{\Phi }}_{i},\;\;\;\;\frac{dP_{I_{i}}}{dt}={\bar{\Phi }}_{i}-{\bar{\Psi }}_{i},\;\;\;\;\frac{dP_{R_{i}}}{dt}={\bar{\Psi }}_{i},$$

(46) $$\frac{dP_{S_{j}S_{i}}}{dt}=-p_{j}\sum_{k}A_{kj}P_{I_{k}S_{j}}P_{S_{i}|S_{j}}+\underset{\left(c\right)}{\underset{︸}{p_{j}A_{ij}P_{I_{i}S_{j}}P_{S_{i}|S_{j}}}}-p_{i}\sum_{k}A_{ki}P_{I_{k}S_{i}}P_{S_{j}|S_{i}}+\underset{\left(d\right)}{\underset{︸}{p_{i}A_{ji}P_{I_{j}S_{i}}P_{S_{j}|S_{i}}}},$$

(47) $$\begin{array}{cc} \frac{dP_{I_{j}S_{i}}}{dt} & =-p_{i}\sum_{k}A_{ki}P_{I_{k}S_{i}}P_{I_{j}|S_{i}}\\ \; & -\underset{\left(a\right)}{\underset{︸}{p_{i}A_{ji}P_{I_{j}S_{i}}\left(1-P_{I_{j}|S_{i}}\right)}}+p_{j}\sum_{k}A_{kj}P_{I_{k}S_{j}}P_{S_{i}|S_{j}}-\underset{\left(b\right)}{\underset{︸}{p_{j}A_{ij}P_{I_{i}S_{j}}P_{S_{i}|S_{j}}}}-q_{j}P_{I_{j}S_{i}}, \end{array}$$

where

(48) $${\bar{\Phi }}_{i}=p_{i}\sum_{j}A_{ij}P_{I_{j}S_{i}},\;\;\;\;{\bar{\Psi }}_{i}=q_{i}P_{I_{i}},\;\;\;P_{S_{j}|S_{i}}=\frac{P_{S_{j}S_{i}}}{P_{S_{i}}},\;\;\;\;P_{I_{j}|S_{i}}=\frac{P_{I_{j}S_{i}}}{P_{S_{i}}}\;.$$

The system involves 5N ordinary differential equations and represents a closed second-order model based on conditional decoupling analogous to those used in conditional methods.

As in the previous subsection, overriding conditional identities (43) by (42) removes the terms (a), (b), (c), and (d) in Equations (46) and (48), which makes these equations coincident with the following identities:

(49) $$\frac{dP_{S_{j}}P_{S_{i}}}{dt}=-p_{j}\sum_{k}A_{kj}P_{I_{k}S_{j}}P_{S_{i}}-p_{i}\sum_{k}A_{ki}P_{I_{k}S_{i}}P_{S_{j}},$$

(50) $$\frac{dP_{I_{j}}P_{S_{i}}}{dt}=-p_{i}\sum_{k}A_{ki}P_{I_{k}S_{i}}P_{I_{j}}+p_{j}\sum_{k}A_{kj}P_{I_{k}S_{j}}P_{S_{i}}-q_{j}P_{I_{j}}P_{S_{i}}\;.$$

This effectively leads to equalities $P_{S_{j}S_{i}}=P_{S_{j}}P_{S_{i}}$ and $P_{I_{j}S_{i}}=P_{I_{j}}P_{S_{i}}$, functionally reducing the conditional second-order closure to the first order.

## 5. Propagation of Epidemic on Simple Graphs

This section investigates the propagation of SIR epidemic on relatively simple algorithmically generated graphs allowing for exact solutions. These results are subsequently compared with the closures.

### 5.1. Exact Solution in One-Dimensional Case

**Figure 1:**
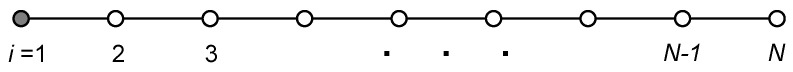
One-dimensional connected graph with initial infection of the first node i=1.

First, we examine the case of one-dimensional propagation of infection, which allows for a relatively simple analytical solution. This is very much analogous to the one-dimensional interpretation used in the original Ising model. Only one connected graph is possible in one dimension that connects the nodes [1,2], [2,3],…,[i,i=1],… that is $A_{i,i\pm 1}=1$ as shown in Figure 1. The initial conditions are specified by

(51) $$Y_{1}=I,\;\;\;Y_{i}=S\;\;\mathrm{for}\;t=0\;\mathrm{and}\;i=2,3,\ldots ,N$$

with a sufficiently large *N*. The probabilities of infection *p* and recovery *q* are deemed to be node-independent constants, that is $p_{i}=p$ and $q_{i}=q$ for all *i*.

We use $P_{iI}$ and $P_{iR}$ to denote the following marginal probabilities:

(52) $$P_{iI}=P\left(I_{i},S_{i+1},\ldots ,S_{N}\right)\;\;\mathrm{and}\;\;P_{iR}=P\left(R_{i},S_{i+1},\ldots ,S_{N}\right)\;,\;$$

where symbols *i*I and *i*R are used as abbreviated notations for the corresponding states

(53) $$iI\overset{\mathrm{def}}{=}\left[Y_{1},\ldots ,Y_{i-1},I_{i},S_{i+1},\ldots ,S_{N}\right]\;\;\;\mathrm{and}\;\;iR\overset{\mathrm{def}}{=}\left[Y_{1},\ldots ,Y_{i-1},R_{i},S_{i+1},\ldots ,S_{N}\right]\;.$$

Note that $Y_{1},\ldots Y_{i-1}$ can be either I or R. These states are subject to the transitions

(54) $${\bar{T}}_{(i+1)I\leftarrow iI}=pP_{iI},\;\;\;{\bar{T}}_{iR\leftarrow iI}=qP_{iI}$$

supplemented by other transitions involving changes in $Y_{1},\ldots ,Y_{i-1}$, which do not need to be considered. The governing equation for probability takes the form

(55) $$\frac{dP_{iI}}{dt}=pP_{(i-1)I}-\left(p+q\right)P_{iI},\;\;\;\frac{dP_{iR}}{dt}=qP_{iI},$$

where i=1,…,N and we formally put $P_{0I}=0$. The one-node probabilities can be easily evaluated from

(56) $$\frac{dP_{S_{i}}}{dt}=-pP_{(i-1)I},\;\;\;\frac{dP_{I_{i}}}{dt}=pP_{(i-1)I}-qP_{I_{i}},\;\;\;\frac{dP_{R_{i}}}{dt}=qP_{I_{i}}\;.$$

### 5.2. Comparison with the Closures

For one-dimensional lattice considered here, Equations (30) and (31), which are associated with the first-order closure, take the form

(57) $$\frac{dP_{S_{i}}}{dt}=-pP_{I_{i-1}}P_{S_{i}},\;\;\;\;\frac{dP_{I_{i}}}{dt}=pP_{I_{i-1}}P_{S_{i}}-qP_{I_{i}},\;\;\;\;\frac{dP_{R_{i}}}{dt}=qP_{I_{i}}\;.$$

These equations are quite different from the exact Equations (55) and (56).

For the second-order direct decoupling closure (37)–(39), the equations for one-node probabilities coincide with (56), assuming $P_{I_{i}S_{i+1}}=P_{iI}$ and $P_{I_{i}S_{i-1}}=0$. The closure equation for the two-node probability $P_{I_{i}S_{i+1}}$

(58) $$\frac{dP_{I_{i}S_{i+1}}}{dt}=pP_{I_{i-1}S_{i}}P_{S_{i+1}}-\left(p+q\right)P_{I_{i}S_{i+1}}$$

is nevertheless different from (55) due to the presence of an additional multiplier, $P_{S_{i+1}},$ in the first term on the right-hand side of Equation (58).

The second-order conditional closure (45)–(48) also reproduces (56), assuming $P_{I_{i}S_{i+1}}=P_{iI}$ and $P_{I_{i}S_{i-1}}=0,$ while the closure equation for the two-node probability

(59) $$\frac{dP_{i}}{dt}=+pP_{i-1}P_{S_{i+1}|S_{i}}-\left(p+q\right)P_{i}$$

is functionally the same as the exact Equation (55) since $P_{S_{i+1}|S_{i}}=1$ under these conditions.

**Figure 2:**
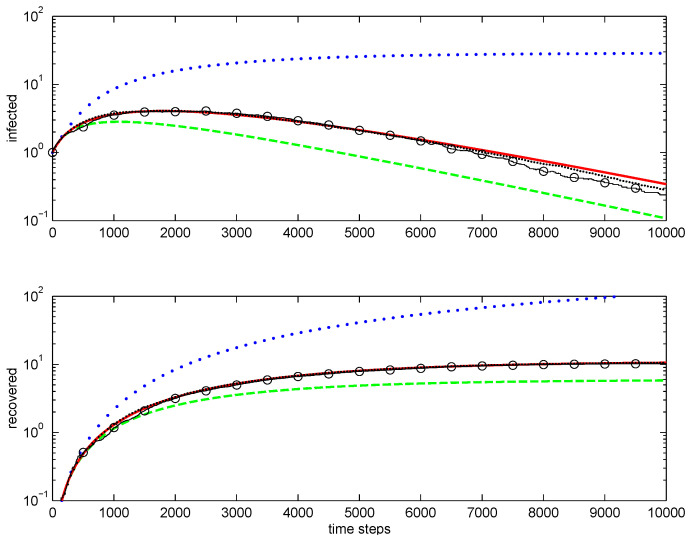
Modelling epidemic in one-dimensional case: total infected (**top figure**) and recovered (**bottom figure**). Lines: ••••, first-order closure; – – – –, second-order direct decoupling closure; —, second-order conditional closure; o—o—o, Monte Carlo ensemble averaging over 100 realisations; ·····, Monte Carlo ensemble averaging over 1000 realisations. Simulation parameters: $\tilde{p}=p\Delta t=5\times 10^{-3},$ $\tilde{q}=q\Delta t=8\times 10^{-4}$.

An example of one-dimensional simulations is shown in Figure 2. The first-order closure does not reproduce correct behaviour. The second-order direct decoupling closure is qualitatively correct but overestimates extinctions. The second-order conditional closure is accurate. Note that, as the average number of infected nodes drops well below unity, averaging becomes difficult for Monte Carlo simulations, since most stochastic realisations do not have any infected nodes.

### 5.3. Epidemic Propagation on a Tree

The first infected node is assigned number 1; each other node is characterised by its number *i* and the distance $l_{i}$ from node 1, which is called level. Obviously, $l_{1}=0$. In a tree, any connected nodes belong to neighbouring levels, that is nodes *i* and *j* can be connected only if $l_{j}=l_{i}\pm 1$. The evolution equations for the marginal probabilities can be obtained from the first-order Equations (21) and (22) and second-order Equations (23) and (24) by taking into account that the graph under consideration is a tree.

Consider three-node probabilities $P_{I_{k}S_{i}Y_{j}}=P($$I_{k},$$S_{i},Y_{j})$ used in (24). Since node *i* is connected to nodes *k* and *j*, there are only two possibilities P($I_{k}^{l-1},$$S_{i}^{l},Y_{j}^{l+1})$ and P($I_{k}^{l+1},$$S_{i}^{l},Y_{j}^{l+1})$ for these probabilities, where $Y_{j}^{l}$ indicates state *Y* of node *j* that belongs to level *l*. It is easy to see that

(60) $$P\left(I_{k}^{l-1},S_{i}^{l},S_{j}^{l+1}\right)\geq 0,\;\;\;\;P\left(I_{k}^{l-1},S_{i}^{l},I_{j}^{l+1}\right)=P\left(I_{k}^{l-1},S_{i}^{l},R_{j}^{l+1}\right)=0$$

and

(61) $$P\left(I_{k}^{l+1},S_{i}^{l},S_{j}^{l+1}\right)=P\left(I_{k}^{l+1},S_{i}^{l},I_{j}^{l+1}\right)=P\left(I_{k}^{l+1},S_{i}^{l},R_{j}^{l+1}\right)=0\;.$$

This implies that all three node correlations of interest are zeros with the exception of P($I_{k}^{l-1},$$S_{i}^{l},$$S_{j}^{l+1}),$ which can be expressed as

(62) $$P\left(I_{k}^{l-1},S_{i}^{l},S_{j}^{l+1}\right)=P\left(I_{k}^{l-1},S_{i}^{l}\right)\;,$$

since $I_{k}^{l-1}$ and $S_{i}^{l}$ always imply $Y_{j}^{l+1}=S$ when nodes *i* and *j* are connected. Substitution of these equalities into (21)–(24) results in the following system:

(63) $$\frac{dP(S_{i}^{l})}{dt}=-{\bar{\Phi }}_{i},\;\;\;\;\frac{dP(I_{i}^{l})}{dt}={\bar{\Phi }}_{i}-{\bar{\Psi }}_{i},\;\;\;\;\frac{dP(R_{i}^{l})}{dt}={\bar{\Psi }}_{i},$$

(64) $${\bar{\Phi }}_{i}=\langle \Phi_{i}\rangle =p_{i}\sum_{k}A_{ji}P\left(I_{j}^{l-1},S_{i}^{l}\right),\;\;\;{\bar{\Psi }}_{i}=\langle \Psi_{i}\rangle =q_{i}P\left(I_{i}^{l}\right),$$

(65) $$\frac{dP(I_{j}^{l},S_{i}^{l+1})}{dt}=p_{i}\sum_{k}A_{ki}P\left(I_{k}^{l-1},S_{j}^{l}\right)-q_{i}P\left(I_{j}^{l},S_{i}^{l+1}\right)\;.$$

This system of equations is closed and does not need any further assumptions. Note that the same equations can be derived from the second-order conditional closure.

**Figure 3:**
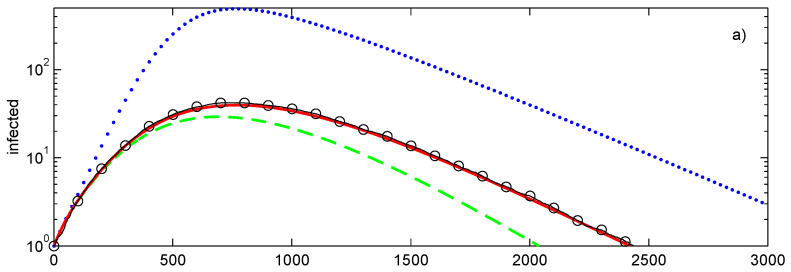

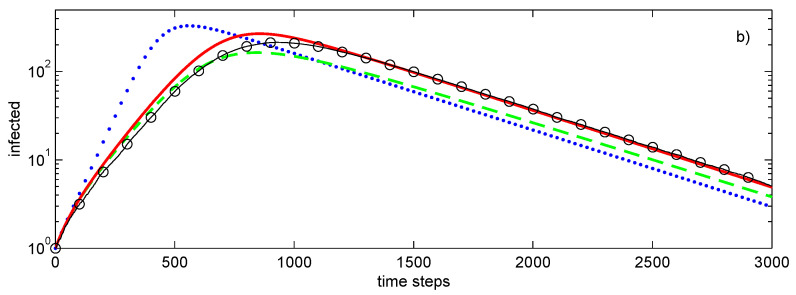
Simulations of SIR epidemic on a tree (**a**), randomly generated graph (**b**) with a fixed degree of $d_{i}=4$ for every node. Lines: ••••, first-order closure; – – – –, second-order direct decoupling closure; —, second-order conditional closure; o—o—o, Monte Carlo ensemble averaging over 100 realisations. Simulation parameters: $\tilde{p}=0.005$, $\tilde{q}=0.003$.

The comparison of the closures with Monte Carlo simulations is shown in Figure 3a. The tree has 1457 nodes in seven layers l=0,…,6. With the exception of the last (seventh) layer, each node has the degree of 4. Epidemic begins at node i=1 located at l=0. As expected, the conditional closure, which is exact in this case, is close to the average of the Monte Carlo simulations. Due to the need of evaluating multiple (100 in this case) realisations, the Monte Carlo simulations require a substantially longer computational time (more than 30 times that of the closures). The second-order decoupling closure has a noticeable error, while the first-order closure is substantially less accurate than the closures of the second order.

## 6. Modelling Epidemic on Scale-Free Networks

The networks used in this section are created with the assistance of random generators, but the solutions are examined here for a fixed typical realisation of each network, i.e., they are not averaged over possible realisations of the networks. As in the previous sections, ensemble averaging implies averaging over realisations of the stochastic simulations of the SIR epidemic on a fixed network. The networks used here are scale-free and possess small-world properties. These networks tend to increase the number of accessible nodes exponentially with each infection transition to neighbours—this matches the initial exponential growth observed in most epidemics. These exponents are strongly affected by the degrees of the nodes involved.

**Figure 4:**
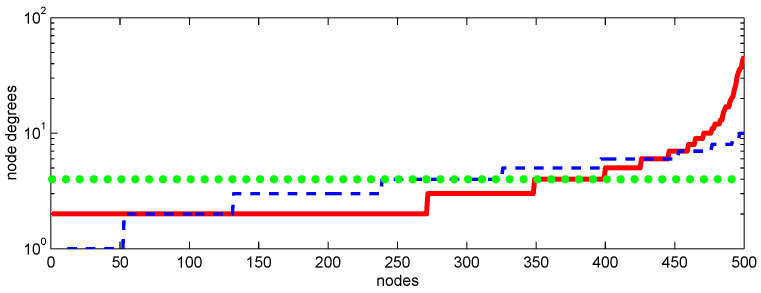
Node degrees versus nodes (ordered by their degrees) for Erdős–Rényi (– – –), Barabasi–Albert (—), and random with fixed degree (••••) graphs used in simulations.

All networks considered in this section have 500 nodes with the average degree of 4 and, as shown in Figure 4, with rather different distributions of node degrees. Figure 3a shows the propagation of the SIR epidemic on a graph with connections between nodes selected at random constrained by the requirement that degree of each node is exactly 4. At the initial stage, this propagation is the same as propagation on a tree graph shown in Figure 3a but as the number of infected nodes increases, the evolutions of these epidemics diverge.

For the Erdos–Rényi graph—the most simple random graph to generate by connecting each couple of nodes with a given probability—the node degrees exhibit some random variations, which have binomial distribution. Another network, which is represented by the Barabási–Albert scale-free graph and, as shown in Figure 4, has the largest variations of the degrees, is considered to give a better representation of the real-world networks. This graph is generated by adding new nodes sequentially with random but preferentially distributed connections proportional to the degrees of the existing nodes. This results in heavy distribution tails: relatively few nodes have many connections. We call these well-connected nodes “central” and the nodes with relatively few connections “peripheral”. While it can be argued that, if compared to real-world networks, the Barabási–Albert graphs tend to overestimate the heaviness of the central nodes, this may be useful since the graphs used in simulations (which have only 150–1500 nodes in the present work) are much smaller than millions of susceptible agents in the real world, and exaggerated clustering of the node degrees in small graphs realistically reflect the concentration of connections in the real-world conditions.

The simulations are performed for the following values of the parameters $\tilde{p}=p\Delta t=0.005$ and $\tilde{q}=q\Delta t=0.003,$ which are assumed to be the same for all nodes. These values are sufficiently small to ensure that simultaneous infection+recovery transitions are unlikely within the same time step. The value of the time step is checked by reducing Δt twice and as expected, this does not affect the results. The time step should be sufficiently small but not too small, as this increases computational expenses. The transmission *p* and recovery *q* probabilities are selected to provide a reasonable value for the q/p ratio ensuring that transmission and recovery have comparable magnitudes for the graphs examined here. The Monte Carlo implementation of the model conventionally generates pseudorandom numbers determining stochastic transitions and, ultimately, the realisations of the process.

**Figure 5:**
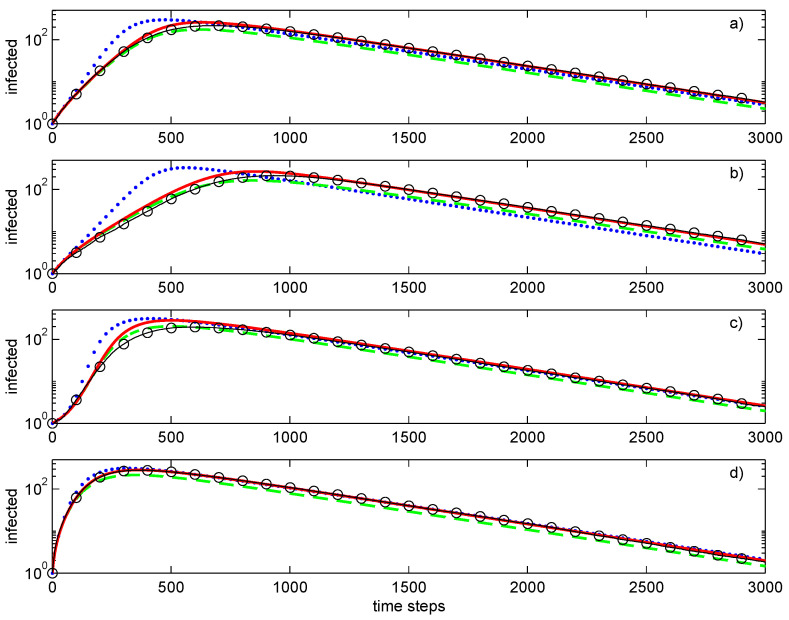
Simulations of SIR epidemic on the Erdős–Rényi (**a**), fixed node degrees (**b**), and Barabasi–Albert (**c**,**d**) graphs with peripheral (**c**) and central (**d**) initial conditions. Lines: ••••, first-order closure; – – – –, second-order direct decoupling closure; —, second-order conditional closure; o—o—o, Monte Carlo ensemble averaging over 100 realisations. Simulation parameters: $\tilde{p}=0.005$, $\tilde{q}=0.003$.

Figure 5 illustrates the outcomes of the simulations. The first-order closure is less accurate than the second-order closures but is still qualitatively correct. The random nature of the graphs tends to increase chaos and decrease correlations between the nodes. Among the second-order closures, the conditional closure is slightly better than the direct decomposition and matches well the averages of stochastic simulations. This averaging is evaluated over 100 independent realisations making Monte Carlo simulations relatively expensive.

The evolutions of the epidemic are substantially different for different networks, even if all of these networks have the same average degree of 4. The fixed degree network has the slowest development of the epidemic and the most stable value of the growth exponent. The epidemic progresses faster for the Barabási–Albert network. The Erdos–Rényi network demonstrates behaviour that is intermediate between that of Barabási–Albert and fixed degree networks. Note that the growth exponent is not constant for networks that have significant variations of the node degrees. This is most evident for the Barabási–Albert network, which demonstrates the largest slope of the exponent followed by its subsequent reduction. This network has another effect associated with the initial conditions: whenever the initial node igniting the epidemic is peripheral, there is a substantial delay in the evolution of the epidemic (as illustrated by Figure 5c in comparison with Figure 5d).

**Figure 6:**
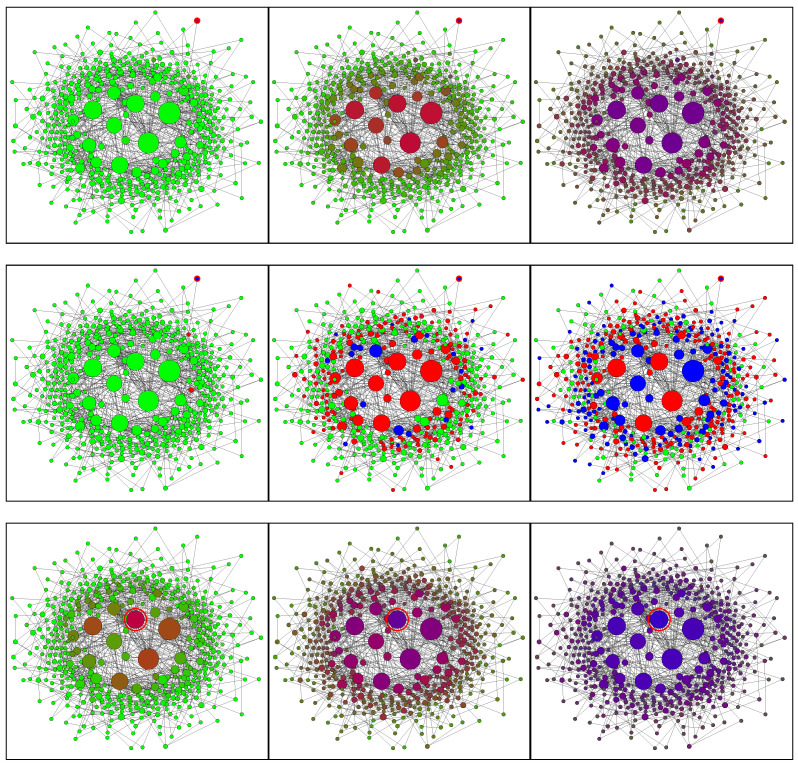

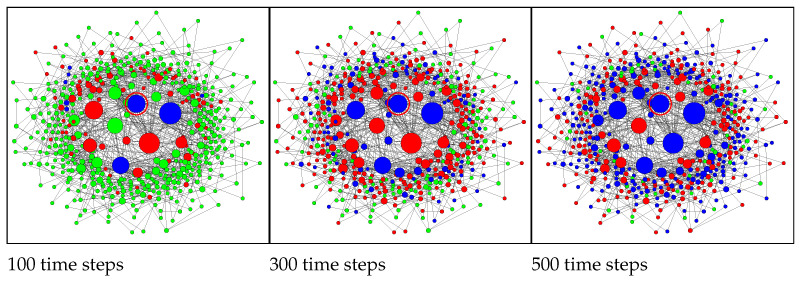
SIR model on the Barabási–Albert network of 500 nodes. Columns from left to right: 100, 300, and 500 time steps. Rows (from top to bottom): 1,2—peripheral initial condition; 3,4—central initial condition; 1,3—conditional closure model; 2,4—Monte Carlo simulations. The initially infected nodes are indicated by the red circles. The node sizes are proportional to the node degrees. Node colours: green—susceptible; red—infected; blue—recovered. Simulation parameters: $\tilde{p}=0.005$, $\tilde{q}=0.003$.

Figure 6 shows the Barabási–Albert network at different stages of the epidemic. Infection of a central node (Figure 6 rows 3,4) leads to fast propagation of the infection to the whole central segment followed by a slower expansion of the epidemic to the peripheral nodes. Initial infection of a peripheral node (Figure 6 rows 1,2) results in a substantial delay in infecting the central segment. During this delay, the epidemic remains latent and, occasionally, might become extinct. Once infection reaches a central node, the rate of infection accelerates dramatically. Note that occasional (local) extinctions amplify correlations between neighbouring nodes and, as this is well-known in the modelling of reacting flows, increase the complexity of the simulations.

## 7. Conclusions

This study evaluates the application of a statistical mechanics-based framework, utilising agent-based Susceptible–Infected–Recovered (SIR) models formulated as continuous-time Markov processes on networks, with the primary aim of testing the effectiveness of combining this approach with complex network geometry. The methodology involves deriving a hierarchical system of marginal probability equations, analogous to the BBGKY hierarchy, to capture complex stochastic transitions and network-driven dependencies within disease spread (although illustrative results are often reported in terms of aggregate averages).

Findings indicate that the second-order conditional closure yields a closed system of equations that approximates Monte Carlo simulations with reasonably high fidelity, particularly in complex topologies, associated with network clustering. The approach can effectively reproduce some notable features of Monte Carlo-simulated epidemic propagation and extinction, as well as the influence of network structure and possible intervention measures, such as lockdowns. The model also appears to reproduce some qualitative aspects of the COVID-19 epidemic in Lombardy: it specifically identified the persistence of infection despite high average herd immunity pointing to the roles of community clustering. However, this application is intended primarily as a methodological assessment—testing the analytical and computational properties of the proposed closure technique—rather than as a comprehensive representation of real-world epidemic processes.

The results further suggest that this statistical mechanics and Markov process approach has potential utility in modelling diffusion phenomena beyond epidemiology, including the adoption of new energy technologies and responses to climate-related disruptions. Future research should address expanded health-state architectures, adaptive network features, and heterogeneous agent behaviours to enhance the model’s capacity for representing multi-phase processes and behavioural feedbacks.

Overall, the study provides evidence supporting the efficacy of hierarchical closure techniques for network-based epidemic modelling, especially at the point when conventional “chaotic” assumptions break and complexity emerges. Its application demonstrates that the framework is applicable to a broad class of diffusion and cascade processes in the presence of complex structures and interlinks. This work establishes a baseline for future methodological refinement and for cross-domain applications of hierarchical modelling of competitive diffusion and emergent complexity in the presence of network heterogeneity and clustering. 

## Figures and Tables

**Figure A1:**
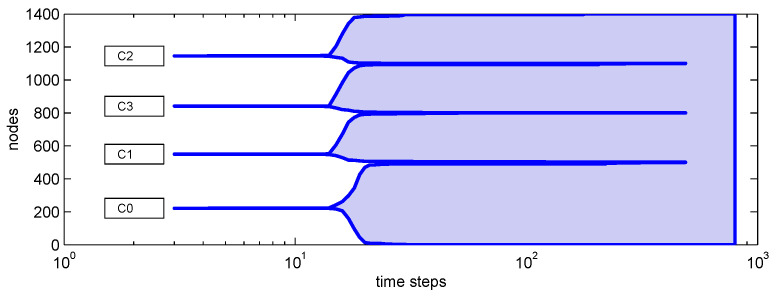
Clustering dendrogram for the composite network.

**Figure A2:**
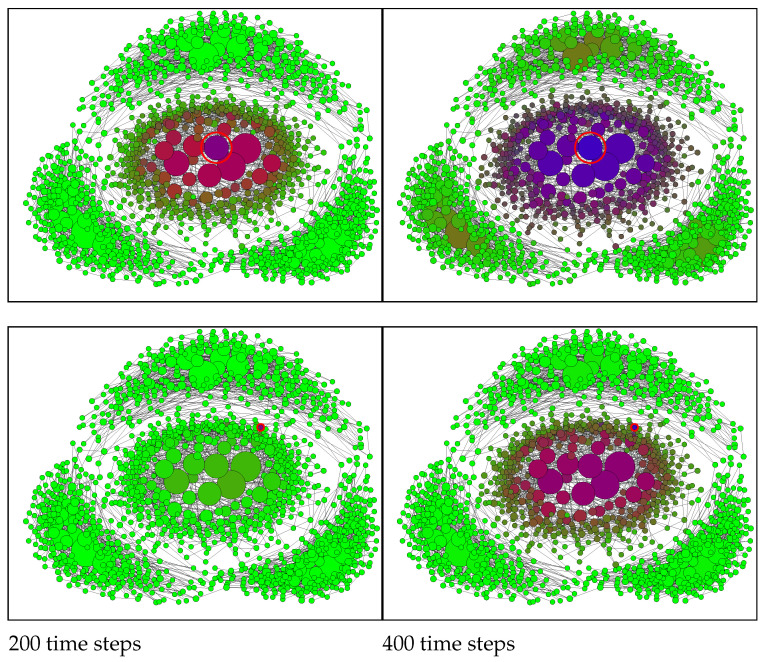
SIR model with the conditional closure on the composite network of four communities at 200 time steps (**left**) and 400 time steps (**right**). The initially infected node is indicated by a red circle and is positioned centrally (**top row**) or peripherally (**bottom row**). The node sizes are proportional to the node degrees. Node colours: green—Susceptible, red—Infected, blue—Recovered. Mixed colours indicate probabilities of S, I, and R. Simulation parameters: $\tilde{p}=0.005$, $\tilde{q}=0.003$.

**Figure A3:**
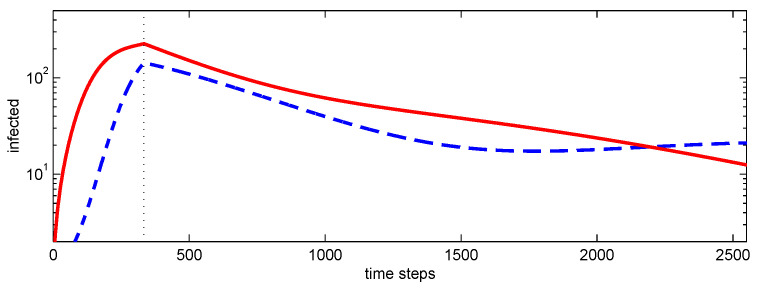
Number of infected nodes versus time step for epidemic simulation using conditional closure and with a lockdown at 333 time steps indicated by the vertical line. Simulation parameters: $\tilde{p}=0.005$ $\tilde{q}=0.0035$, while $\tilde{p}=0.001$ after lockdown. Initial condition: (a) central (—), (b) peripheral (– – –).

**Figure A4:**
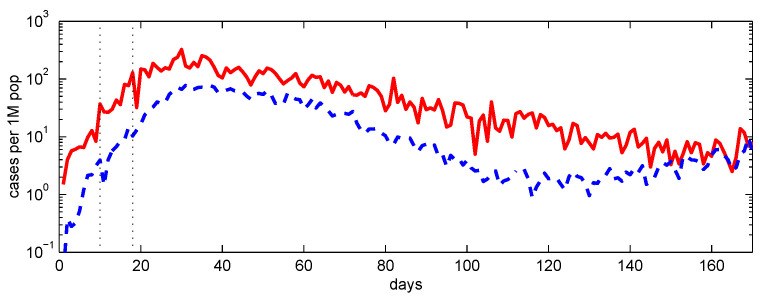
COVID-19 infections per 1 million population in Italy during the first COVID-19 wave, February–June 2020. Lines (a) — Lombardy, (b) – – – the rest of Italy (excluding Lombardy). The vertical lines indicate the lockdown time and the effective delay associated with the latent period.

## Data Availability

Data is contained within the article.

## Nomenclature

$Y_{i}\in \left\{S,I,R\right\}$	SIR states
$Y^{\left(N\right)}=Y_{1},\ldots ,Y_{N}$	System state vector
$P_{Y}$	Full joint probability
〈·〉	Ensemble average
$\theta_{i}\left(Y_{i}^{\circ }\right)=\delta_{Y_{i}Y_{i}^{\circ }}\in \left\{0,1\right\}$	Indicator function
$f^{\left(n\right)}=\theta_{i_{1}}\left(\cdot \right)\cdots \theta_{i_{n}}\left(\cdot \right)$	Fine-grained distribution
$P^{\left(n\right)}=\langle f^{\left(n\right)}\rangle =\langle \theta_{i_{1}}\cdots \theta_{i_{n}}\rangle$	Marginal probability
$A_{ij}$, with $A_{ij}=A_{ji}$ and $A_{ii}=0$	Adjacency matrix
*T*	Transition-rate operator
S→I→R	Infection/recovery transitions
$p_{i}$	Infection parameter
$q_{i}$	Recovery parameter
$\Phi_{i}=\sum_{j}p_{i}A_{ij}\;\theta_{i}\left(S\right)\;\theta_{j}\left(I\right)$	Infection transition rate (operator)
$\Psi_{i}=q_{i}\;\theta_{i}\left(I\right)$	Recovery transition rate (operator)
${\bar{\Phi }}_{i},\;{\bar{\Psi }}_{i}$	Averaged transition rates

## Appendix A. Network Clustering and Epidemic Waves

Real-world contact networks are rarely homogeneous: they typically exhibit community structure (clusters) and, often, a hierarchy of subclusters. Such clustering can strongly modify epidemic dynamics and may generate multi-wave behaviour even in simple agent-based SIR settings on static graphs; see, e.g., network-based studies in computational epidemiology [20,21,22]. In particular, once the infection has largely saturated the central (high-degree) nodes of one community and local herd immunity begins to form, the epidemic may nevertheless persist if the infection subsequently reaches the central nodes of other communities. This mechanism provides a natural route to prolonged tails and secondary waves [23]. Note that communities is a mathematical term describing clustering in networks. Such communities may coincide with the everyday meaning of the term (here we refer to overt communities, such as those associated with geographical location), but they may also be latent—not physically separated and not directly observable. Consequently, transmission between communities may or may not be evident in practice, and an intrinsically inhomogeneous process can appear homogeneous in aggregate data.

These subtle divisions between communities may become more pronounced in modern societies as people are better informed and adjust their behaviour, while governments introduce emergency regulations. Measures commonly described as “lockdowns” can be represented, at a minimal level, by a reduction in the effective propagation probability *p* and/or by reduced inter-community mixing; in clustered networks, this can temporarily delay spill-over between communities but does not, by itself, eliminate the possibility of later re-amplification.

To illustrate this effect, we consider a composite network formed by four Barabási–Albert graphs: one principal community (500 nodes) and three secondary communities (300 nodes each), weakly connected so that their central segments do not merge. The clustering dendrogram in Figure A1 confirms the presence of four communities [24]. Figure A2 shows a representative evolution in which the epidemic first infects the principal community (“metropolis”) and begins to subside there as immunity accumulates, yet escapes into secondary communities and produces renewed growth. Figure A3 compares central and peripheral initial conditions on the same clustered network and demonstrates that the post-intervention evolution can be governed by competing trends: attenuation within the initially affected community versus delayed ignition of other communities, which can generate secondary waves or long-lasting plateau.

A qualitatively similar pattern may have been present during the first wave of the COVID-19 epidemic in Italy. As illustrated in Figure A4, the decline in Lombardy appears slower than in neighbouring regions despite a larger initial burden [25] and, therefore, presumably higher immunity, at least in some segments of the population. This observation is not straightforward to reconcile with a single well-mixed community, where deeper penetration of the infection would typically be expected to accelerate the subsequent decay.

While many factors may contribute, clustered network dynamics provide a natural explanation. Strong early spread within one community—potentially triggered by an unfortunate early infection of highly connected (central) individuals—can coexist with delayed propagation into other communities. Deep penetration then has two competing effects: it builds immunity within the initially affected community, while also seeding infection more widely across other communities. As incidence declines in the first community owing to local herd immunity, transmission initiated elsewhere can sustain the overall level of infection, producing persistence and a prolonged tail without necessarily developing into a distinct second wave. The qualitative similarity between the trajectories in Figure A3 and Figure A4 supports the plausibility of this interpretation.
